# The potential of BRD4 inhibition in tumour mechanosignaling

**DOI:** 10.1111/jcmm.18057

**Published:** 2023-11-23

**Authors:** Antonios N. Gargalionis, Kostas A. Papavassiliou, Athanasios G. Papavassiliou

**Affiliations:** ^1^ Department of Biopathology, ‘Eginition’ Hospital, Medical School National and Kapodistrian University of Athens Athens Greece; ^2^ First University Department of Respiratory Medicine, ‘Sotiria’ Hospital, Medical School National and Kapodistrian University of Athens Athens Greece; ^3^ Department of Biological Chemistry, Medical School National and Kapodistrian University of Athens Athens Greece

**Keywords:** BRD4, mechanosignaling, targeted therapy, TAZ, tumour inhibitor, YAP

It is broadly acknowledged that physical properties of cancer cells play a crucial role in all aspects of tumour development, from cancer initiation to malignant progression. Aberrant mechanical forces shape a dynamic interplay between tumour cells, extracellular matrix (ECM) and cells of the tumour microenvironment to promote cancer cell proliferation, invasion, metastasis and drug resistance. To this end, recent research efforts are focusing on therapeutically targeting the mechanical hallmarks of cancer and exploiting these properties for improved clinical outcomes.^1^ Well‐established mechanosensitive molecules mediate this interplay, thereby configurating the processes of mechanotransduction. These mediators encompass membrane receptors such as integrins, downstream kinase pathways [e.g. the focal adhesion kinase (FAK) signalling pathway] and transcriptional effectors, dominantly the Yes‐associated protein 1 (YAP1) and transcriptional coactivator with PDZ‐binding motif (TAZ) complex. YAP/TAZ are the terminal effectors of the Hippo signal transduction cascade, which is involved in ECM alterations to regulate cell proliferation, self‐maintenance of stem cells and organ development. YAP/TAZ trigger the expression of tumour‐promoting genes and are implicated in mechanisms of drug resistance.^2^, ^3^, ^4^

It has been documented that the physical interaction of the YAP/TAZ complex with the chromatin‐binding histone acetyltransferase bromodomain‐containing protein 4 (BRD4) is essential to perform this genomic action. BRD4, a well‐studied member of the bromodomain and extraterminal domain (BET) protein family, is expressed in a wide range of somatic cells across tissues. It plays an important role in various physiological processes as demonstrated in mouse studies where *BRD4*‐null homozygotes do not survive after implantation and *BRD4*‐null heterozygotes display abnormal growth and several structural deformities.^5^ Mechanistically, BRD4 functions as a transcriptional coactivator and is recruited by YAP/TAZ to acetylate histones H3 and H4, especially promoting the histone mark H3K122ac. Formation of this complex recruits, in turn, RNA polymerase II to enhance the transcriptional activity of the YAP1/TAZ target proliferation/cell cycle progression‐controlling genes, as well as to dictate further mechanosensing‐related transcriptional events.^2^, ^6^, ^7^

The YAP/TAZ/BRD4 complex participates in tumour progression, angiogenesis, metastasis and resistance to therapy.^2^, ^4^ YAP/TAZ mediate transcriptional addiction in cancer cells, meaning that YAP/TAZ‐dependent gene expression is enriched in tumour cells. To achieve this, when YAP/TAZ are bound to enhancers, they facilitate the recruitment of BRD4 on target promoters, ultimately resulting in increased expression of critical genes. BRD4 inhibition by BET inhibitors (BETi) has been shown to effectively suppress downstream gene transcription even when YAP is overexpressed, suggesting that YAP/TAZ function upstream of BRD4. Conversely, BRD4's overexpression fails to induce the expression of YAP/TAZ target genes when YAP/TAZ are absent, demonstrating the tight reciprocal interplay between these molecular factors of the transcriptional machinery.^7^ In oesophageal adenocarcinoma (EAC), BRD4 has been found to directly bind to the promoter of *YAP1*. YAP1 expression and that of its target genes, such as *connective tissue growth factor* (*CTGF*), *SOX9* and *cysteine‐rich angiogenic inducer 61* (*Cyr61*), is downregulated when BET proteins are targeted in EAC cells. BRD4 inhibition also suppresses cancer stem cell properties and decreases respective aldehyde dehydrogenase 1 (ALDH1) + marked stem cells in radiation‐resistant EAC cells. This effect is augmented following the addition of docetaxel in vitro and in vivo.^8^ Furthermore, BRD4 is capable of tethering to *collagen‐ and calcium‐binding epidermal growth factor domain‐containing protein 1* (*CCBE1*) enhancers, a known booster of tumour lymphangiogenesis. This interaction is sustained even when mechanosensitive YAP/TAZ are absent, implying that there is a mechanism for BRD4‐mediated modulation of *CCBE1* transcription, which is independent of the YAP/TAZ‐associated transcriptional enhancer factor TEF‐3 (TEA domain family member 4, TEAD4) complex.^9^ In head and neck squamous cell carcinoma (HNSCC), BRD4 interacts with the YAP1/TAZ/TEAD transcriptional complex to facilitate expression of oncogenes. FAT atypical cadherin 1 (FAT1) is an upstream inhibitor of YAP1, and its gene harbours frequent mutations in HNSCC. *FAT1* mutations are linked to increased nuclear expression of YAP1. Additionally, suppression of *FAT1* expression upregulates the YAP1 transcriptional program and, specifically, the transcription of YAP1 target genes such as *CTGF*, *Cyr61* and *ankyrin repeat domain‐containing protein 1* (*ANKRD1*). HNSCC with *FAT1* mutations presents increased sensitivity to BRD4 inhibition, thus offering a potential predictive marker for a new therapeutic approach.^10^ BRD4 and YAP1 also interact in human pancreatic ductal adenocarcinoma to stimulate the expression of receptor tyrosine kinase‐like orphan receptor 1 (ROR1). ROR1 is highly expressed in cells exhibiting traits of epithelial‐to‐mesenchymal transition (EMT). Upon BRD4/YAP targeting, ROR1 expression is reduced, thereby preventing cancer cell growth.^11^ Moreover, BETi display encouraging effects in uveal melanomas (UMs). Unlike BRAF‐mutated cutaneous melanomas (CMs), UMs are generated from somatic activating mutations in *guanine nucleotide‐binding protein G*(*q*) *subunit alpha* (*GNAQ*) or *guanine nucleotide‐binding protein subunit alpha‐11* (*GNA11*), encoding Gq or G11, respectively. BETi are effective in Gq‐mutated UMs by targeting BRD4, hence suppressing YAP expression. However, when YAP/TAZ are downregulated, transcription of BRD4 is reduced only in Gq‐mutated cells. This mutual interaction explains BRD4 effective inhibition in Gq‐mutated UMs compared to BRAF‐mutated CM cells and animals. Consequently, there are potential clinical implications of BRD4 inhibition in Gq‐mutated cancer cell subpopulation.^12^ In non‐small cell lung cancer (NSCLC), the combination of the small BETi PLX51107 with RAS, RAF or mitogen‐activated protein kinase (MEK) inhibitors represses cell proliferation, cell viability, induces apoptosis and downregulates *myc* transcription in vitro in BRAF and KRAS‐mutated cells. The inhibitory combination also suppresses tumour growth in vivo in cell line‐ and patient‐derived xenografts. Nevertheless, when BRAF and KRAS mutations coexist with mutations of the tumour suppressor serine/threonine kinase 11 (STK11; also known as liver kinase B1, LKB1), cancer cells develop resistance to this dual inhibition, suggesting a new predictive biomarker to tailor combinatorial treatment.^13^

BETi are low‐molecular‐mass compounds which can target either one of the two [bromodomain 1 (BD1) or bromodomain 2 (BD2)] or both bromodomains (pan‐BET bromodomain inhibitors).^14^, ^15^ BETi also have the ability to reduce tumour burden selectively by targeting super‐enhancers and, thereby, reduce the transcription of multiple oncogenes.^16^ JQ1 is the most frequently tested BETi, demonstrating the highest binding affinity for BRD4 among all human proteins containing bromodomains.^14^, ^15^, ^17^ Preclinical studies highlight a multitude of developed BETi as promising therapeutic agents in a broad spectrum of malignancies, with several inhibitors progressing to clinical trials either as monotherapy or in combinatorial regimens.^14^, ^15^ Dose‐limiting toxicities emerge such as anaemia, neutropenia and gastrointestinal manifestations.^18^ Notably, however, compared with their antagonistic antitumor histone deacetylase (HDAC) inhibitors (e.g. FK228), which present with a wide range of direct cytotoxicities and can cause various immune alterations via the expression of costimulatory molecules [e.g. programmed death‐ligand 1 (PD‐L1), major histocompatibility complex (MHC), tumour antigens and cytokines],^19^, ^20^ BETi display mainly thrombocytopenia as the most common dose‐limiting adverse effect in cancer patients on monotherapy.^21^

A number of clinical studies evaluating BETi in various clinical settings are currently in progress and/or have been completed (https://clinicaltrials.gov/).^14^, ^15^, ^22^ Besides cancer, apabetalone (RVX‐208) has progressed in a phase 3 trial against heart failure following an acute coronary syndrome.^23^ In the MANIFEST‐2 study, pelabresil (CPI‐0610) is a small molecule/BETi that is being tested in combination with ruxolitinib [a Janus kinase (JAK) inhibitor] versus placebo plus ruxolitinib in patients with myelofibrosis who have never received treatment with JAK inhibitors. Preliminary results showed significant decrease in spleen volume and total symptom score with the study advancing to phase 3 trial.^18^, ^24^ Pelabresil has been also assessed as monotherapy in phase 1 trials regarding multiple myeloma and progressive lymphoma.^25^ BMS‐986158 is a selective BETi that is being tested in phase 1 trials as monotherapy for paediatric cancer and as monotherapy or in combination with nivolumab (anti‐PD‐1) in selected advanced tumours.^26^ ZEN3694 is being evaluated in phase 1 trials for patients with solid tumours in several combinatorial regimens, such as with nivolumab with or without ipilimumab [anti‐cytotoxic T‐lymphocyte‐associated protein 4 (CTLA‐4), also known as CD152], for adult/paediatric patients treated with abemaciclib [cyclin‐dependent kinase (CDK) inhibitor] or chemotherapy and those treated with entinostat (HDAC inhibitor). It is also evaluated in combination with binimetinib (MEK inhibitor) in patients suffering from solid tumours with RAS alterations and in triple‐negative breast cancer (TNBC), as well as with a PD‐1 inhibitor (pembrolizumab) and standard chemotherapy in patients with advanced TNBC. In addition, it has been employed in phase 2 trials for patients with solid tumours and recurrent ovarian cancer treated with talazoparib [poly (ADP‐ribose) polymerase (PARP) inhibitor], for patients with metastatic castration‐resistant prostate cancer treated with enzalutamide (androgen receptor inhibitor) and pembrolizumab^27^ and as monotherapy for patients with squamous cell lung cancer. Molibresib (GSK525762) and birabresib (MK‐8628/OTX015) have been assessed in phase 1/2 studies for patients with relapsed, refractory hematologic malignancies and solid tumours exhibiting potent anticancer activity; however, there were significant toxicities limiting their use.^28^, ^29^, ^30^ Further small molecule/BETi that are currently being evaluated in phase 1/2 trials include TQB3617 in patients with advanced tumours and NUV‐868 as monotherapy and in combination with olaparib (PARP inhibitor) or enzalutamide in adult patients with advanced solid tumours (https://clinicaltrials.gov/).

According to the data provided above, application of BETi and YAP/TAZ/TEAD targeting in preclinical studies demonstrates potential antitumor efficacy.^4^, ^14^, ^15^ Nonetheless, both BET and YAP/TAZ inhibition display limited efficacy as monotherapies and seem to be more effective when they are administered in combinatorial therapies overcoming multiple resistance mechanisms. Given the reciprocal activity of BRD4 with the YAP/TAZ complex, dual BRD4 and YAP/TAZ inhibition could potentially enhance therapeutic efficacy with respect to mechanosignaling‐associated properties of tumour cells. Experimental data reveal that BRD4 inhibition can bridle YAP/TAZ overexpression and lead to YAP/TAZ/BRD4‐associated suppression of gene transcription. Therefore, administration of BETi should be evaluated in preclinical and clinical studies to overcome resistance or inefficacy of YAP/TAZ/TEAD inhibitors.^7^ As a result of the fact that BRD4 demonstrates YAP/TAZ‐independent transcriptional activity, combined YAP/TAZ/BRD4 targeting could also address the full extent of the tumour mechanosignaling‐engaged impact on cancer progression.^9^ As a proof of concept, preclinical data highlight the efficacy of BRD4 inhibition when YAP/TAZ/TEAD negative regulators lose their function, implying that BRD4 suppression could be an alternative therapeutic strategy to diminish YAP/TAZ‐induced gene expression.^10^

In conclusion, although the application of BETi in the clinic seems promising, their full potential has not yet been realized due to the lack of satisfactory predictive biomarkers. Additionally, these pharmacological agents exhibit some notable dose‐limiting toxicities, ultimately abating their antitumor effects. The therapeutic potential of BRD4 inhibition lies in combinatorial treatments and dual BRD4 and YAP/TAZ/TEAD inhibition could be a rational therapeutic approach to improve clinical outcome. To this end, it is important to further clarify the molecular mechanisms underpinning BRD4 and YAP/TAZ interactions in various preclinical and clinical settings and identify potential predictive biomarkers and alternative routes to overcome resistance to these chemical modalities.

## AUTHOR CONTRIBUTIONS

**Antonios N. Gargalionis:** Conceptualization (equal); data curation (equal); writing – original draft (lead). **Kostas A. Papavassiliou:** Conceptualization (equal); data curation (equal); writing – original draft (equal). **Athanasios G. Papavassiliou:** Conceptualization (lead); data curation (lead); supervision (lead); writing – review and editing (lead).

## CONFLICT OF INTEREST STATEMENT

The authors declare no competing financial interests.

## Data Availability

Data sharing not applicable‐no new data generated.

## References

[1] Gargalionis AN , Basdra EK , Papavassiliou AG . Mechanosignalling in tumour progression. J Cell Mol Med. 2018;22(2):704‐705. doi:10.1111/jcmm.13452 29134745 PMC5783856

[2] Battilana G , Zanconato F , Piccolo S . Mechanisms of YAP/TAZ transcriptional control. Cell Stress. 2021;5(11):167‐172. doi:10.15698/cst2021.11.258 34782888 PMC8561301

[3] Dupont S , Morsut L , Aragona M , et al. Role of YAP/TAZ in mechanotransduction. Nature. 2011;474(7350):179‐183. doi:10.1038/nature10137 21654799

[4] Gargalionis AN , Papavassiliou KA , Papavassiliou AG . Targeting the YAP/TAZ mechanotransducers in solid tumour therapeutics. J Cell Mol Med. 2023;27(13):1911‐1914. doi:10.1111/jcmm.17794 37226849 PMC10315712

[5] Houzelstein D , Bullock SL , Lynch DE , Grigorieva EF , Wilson VA , Beddington RS . Growth and early postimplantation defects in mice deficient for the bromodomain‐containing protein Brd4. Mol Cell Biol. 2002;22(11):3794‐3802. doi:10.1128/MCB.22.11.3794-3802.2002 11997514 PMC133820

[6] Alam J , Huda MN , Tackett AJ , Miah S . Oncogenic signaling‐mediated regulation of chromatin during tumorigenesis. Cancer Metastasis Rev. 2023;42(2):409‐425. doi:10.1007/s10555-023-10104-3 37147457 PMC10348982

[7] Zanconato F , Battilana G , Forcato M , et al. Transcriptional addiction in cancer cells is mediated by YAP/TAZ through BRD4. Nat Med. 2018;24(10):1599‐1610. doi:10.1038/s41591-018-0158-8 30224758 PMC6181206

[8] Song S , Li Y , Xu Y , et al. Targeting Hippo coactivator YAP1 through BET bromodomain inhibition in esophageal adenocarcinoma. Mol Oncol. 2020;14(6):1410‐1426. doi:10.1002/1878-0261.12667 32175692 PMC7266288

[9] Song J , Dang X , Shen X , et al. The YAP‐TEAD4 complex promotes tumor lymphangiogenesis by transcriptionally upregulating CCBE1 in colorectal cancer. J Biol Chem. 2023;299(4):103012. doi:10.1016/j.jbc.2023.103012 36781122 PMC10124907

[10] Chen N , Golczer G , Ghose S , et al. YAP1 maintains active chromatin state in head and neck squamous cell carcinomas that promotes tumorigenesis through cooperation with BRD4. Cell Rep. 2022;39(11):110970. doi:10.1016/j.celrep.2022.110970 35705032

[11] Yamazaki M , Hino S , Usuki S , et al. YAP/BRD4‐controlled ROR1 promotes tumor‐initiating cells and hyperproliferation in pancreatic cancer. EMBO J. 2023;42(14):e112614. doi:10.15252/embj.2022112614 37096681 PMC10350825

[12] Zhang GM , Huang SS , Ye LX , et al. Reciprocal positive regulation between BRD4 and YAP in GNAQ‐mutant uveal melanoma cells confers sensitivity to BET inhibitors. Pharmacol Res. 2022;184:106464. doi:10.1016/j.phrs.2022.106464 36162600

[13] Chatterjee N , Olivas V , Wu W , Powell B , Bivona T . Targeting Hippo‐YAP, BRD4 and RAS‐MAPK interplay in lung cancer to forestall drug resistance. Cancer Res. 2023;83(7_Suppl):3878.

[14] Shorstova T , Foulkes WD , Witcher M . Achieving clinical success with BET inhibitors as anti‐cancer agents. Br J Cancer. 2021;124(9):1478‐1490. doi:10.1038/s41416-021-01321-0 33723398 PMC8076232

[15] Trojer P , Targeting BET . Bromodomains in cancer. Annu Rev Cancer Biol. 2022;6(1):313‐336. doi:10.1146/annurev-cancerbio-070120-103531

[16] Chen YL , Li XL , Li G , et al. BRD4 inhibitor GNE987 exerts anti‐cancer effects by targeting super‐enhancers in neuroblastoma. Cell Biosci. 2022;12(1):33. doi:10.1186/s13578-022-00769-8 35303940 PMC8932231

[17] Filippakopoulos P , Qi J , Picaud S , et al. Selective inhibition of BET bromodomains. Nature. 2010;468(7327):1067‐1073. doi:10.1038/nature09504 20871596 PMC3010259

[18] Battaglia G . BET inhibitors in cancer therapy: finding the right combination. Onc Live. 2023;24(5):33‐35.

[19] Konstantinopoulos PA , Vandoros GP , Papavassiliou AG . FK228 (depsipeptide): a HDAC inhibitor with pleiotropic antitumor activities. Cancer Chemother Pharmacol. 2006;58(5):711‐715. doi:10.1007/s00280-005-0182-5 16435156

[20] Shi Y , Fu Y , Zhang X , et al. Romidepsin (FK228) regulates the expression of the immune checkpoint ligand PD‐L1 and suppresses cellular immune functions in colon cancer. Cancer Immunol Immunother. 2021;70(1):61‐73. doi:10.1007/s00262-020-02653-1 32632663 PMC7838139

[21] Sun Y , Han J , Wang Z , Li X , Sun Y , Hu Z . Safety and efficacy of bromodomain and extra‐terminal inhibitors for the treatment of hematological malignancies and solid tumors: a systematic study of clinical trials. Front Pharmacol. 2020;11:621093. doi:10.3389/fphar.2020.621093 33574760 PMC7870522

[22] Wu D , Qiu Y , Jiao Y , Qiu Z , Liu D . Small molecules Targeting HATs, HDACs, and BRDs in cancer therapy. Front Oncol. 2020;10:560487. doi:10.3389/fonc.2020.560487 33262941 PMC7686570

[23] Nicholls SJ , Schwartz GG , Buhr KA , et al. Apabetalone and hospitalization for heart failure in patients following an acute coronary syndrome: a prespecified analysis of the BETonMACE study. Cardiovasc Diabetol. 2021;20(1):13. doi:10.1186/s12933-020-01199-x 33413345 PMC7791841

[24] Harrison CN , Gupta VK , Gerds AT , et al. Phase III MANIFEST‐2: pelabresib + ruxolitinib vs placebo + ruxolitinib in JAK inhibitor treatment‐naive myelofibrosis. Future Oncol. 2022;18(27):2987‐2997. doi:10.2217/fon-2022-0484 35950489

[25] Blum KA , Supko JG , Maris MB , et al. A phase I study of pelabresib (CPI‐0610), a small‐molecule inhibitor of BET proteins, in patients with relapsed or refractory lymphoma. Cancer Res Commun. 2022;2(8):795‐805. doi:10.1158/2767-9764.CRC-22-0060 36923307 PMC10010313

[26] Hilton J , Cristea M , Postel‐Vinay S , et al. BMS‐986158, a small molecule inhibitor of the bromodomain and extraterminal domain proteins, in patients with selected advanced solid tumors: results from a phase 1/2a trial. Cancers (Basel). 2022;14(17):4079. doi:10.3390/cancers14174079 36077617 PMC9454848

[27] Aggarwal RR , Schweizer MT , Nanus DM , et al. A phase Ib/IIa study of the Pan‐BET inhibitor ZEN‐3694 in combination with enzalutamide in patients with metastatic castration‐resistant prostate cancer. Clin Cancer Res. 2020;26(20):5338‐5347. doi:10.1158/1078-0432.CCR-20-1707 32694156 PMC7572827

[28] Dawson MA , Borthakur G , Huntly BJP , et al. A phase I/II open‐label study of molibresib for the treatment of relapsed/refractory hematologic malignancies. Clin Cancer Res. 2023;29(4):711‐722. doi:10.1158/1078-0432.CCR-22-1284 36350312 PMC9932578

[29] Cousin S , Blay JY , Garcia IB , et al. Safety, pharmacokinetic, pharmacodynamic and clinical activity of molibresib for the treatment of nuclear protein in testis carcinoma and other cancers: results of a phase I/II open‐label, dose escalation study. Int J Cancer. 2022;150(6):993‐1006. doi:10.1002/ijc.33861 34724226

[30] Lewin J , Soria JC , Stathis A , et al. Phase Ib trial with birabresib, a Small‐molecule inhibitor of bromodomain and extraterminal proteins, in patients with selected advanced solid tumors. J Clin Oncol. 2018;36(30):3007‐3014. doi:10.1200/JCO.2018.78.2292 29733771

